# Shoot, shovel and shut up: cryptic poaching slows restoration of a large carnivore in Europe

**DOI:** 10.1098/rspb.2011.1275

**Published:** 2011-08-17

**Authors:** Olof Liberg, Guillaume Chapron, Petter Wabakken, Hans Christian Pedersen, N. Thompson Hobbs, Håkan Sand

**Affiliations:** 1 Grimsö Wildlife Research Station, Department of Ecology, Swedish University of Agricultural Sciences, 73091 Riddarhyttan, Sweden; 2 Department of Applied Ecology and Agricultural Sciences, Hedmark University College, Evenstad 2480, Koppang, Norway; 3 Norwegian Institute for Nature Research, Tungasletta 2, 7485 Trondheim, Norway; 4 Natural Resource Ecology Laboratory and Graduate Degree Program in Ecology, Colorado State University, Fort Collins, CO 80523, USA

**Keywords:** state–space models, poaching, wolf, *Canis lupus*, conservation

## Abstract

Poaching is a widespread and well-appreciated problem for the conservation of many threatened species. Because poaching is illegal, there is strong incentive for poachers to conceal their activities, and consequently, little data on the effects of poaching on population dynamics are available. Quantifying poaching mortality should be a required knowledge when developing conservation plans for endangered species but is hampered by methodological challenges. We show that rigorous estimates of the effects of poaching relative to other sources of mortality can be obtained with a hierarchical state–space model combined with multiple sources of data. Using the Scandinavian wolf (*Canis lupus*) population as an illustrative example, we show that poaching accounted for approximately half of total mortality and more than two-thirds of total poaching remained undetected by conventional methods, a source of mortality we term as ‘cryptic poaching’. Our simulations suggest that without poaching during the past decade, the population would have been almost four times as large in 2009. Such a severe impact of poaching on population recovery may be widespread among large carnivores. We believe that conservation strategies for large carnivores considering only observed data may not be adequate and should be revised by including and quantifying cryptic poaching.

## 1. Introduction

The illegal killing of animals, hereafter poaching, threatens the viability of many species worldwide [1–5]. Because of their characteristic low densities combined with their slow rates of population growth, top predators are particularly vulnerable to effects of poaching. Almost all large carnivore species have endured a long history of human persecution and have been eradicated from substantial parts of their historical ranges [6]. Although most species of large carnivores are now legally protected, poaching remains a widespread problem for their conservation [6]. Some species are commercially poached for pelts or body parts used in traditional medicine [7], but many are killed because of conflicts with human interests, such as competition for game, depredation of livestock and threats to human safety [8]. It follows that dealing with poaching mortality often emerges as a required condition for the restoration, conservation and sustainable management of large carnivore populations.

A near universal problem with understanding poaching is the absence of rigorous estimates of its effects relative to other sources of mortality [1]. There are several recent attempts to assess the extent, mechanisms and effects of poaching [2,9–12] but remarkably little quantitative data exist, although new methods to measure its extent have recently been developed [13]. One obvious reason for the absence of data is methodological. The most reliable method of quantifying causes of mortality in populations of large wild mammals is to observe their fates over time using radio-tracking [14]. However, when a radio-collared animal is poached, there is a high probability that the poacher promptly destroys the transmitter and hides (or consumes) the carcass, leaving the researcher with a lost radio contact without known cause [15]. Treating cases of lost radio contact in a survival analysis based on radio-tracking is not a trivial problem, especially not for such ‘poaching-prone’ animals as large carnivores. One can never exclude the possibility that a certain proportion of animals with lost radio contact in fact died from poaching that cannot be verified. We define this unobserved source of mortality as ‘cryptic poaching’. Estimating a quantity in ecological processes that is not amenable to direct observation is feasible with hierarchical models because these models allow multiple sources of data to inform estimates of model parameters, including unobservable ones [16]. These sources of data can include observations on state variables from monitoring studies as well as direct estimates of observable parameters from detailed studies of processes. In this paper, we used a decade (1999–2009) of population census, radio-tracking and recruitment data of the Scandinavian wolf population combined with a Bayesian state–space hierarchical population model to show that poaching has drastically slowed down the recovery of this population.

## 2. Material and methods

### (a) General approach

Between December 1998 and April 2009, we radio-marked 104 wolves in Scandinavia constituting between 10 and 15 per cent of the population, among which we had 26 verified mortalities. We used radio-tracking data to compute three cause-specific mortality rates based on 21 cases of non-poaching (seven natural deaths such as age and disease, five traffic mortalities and nine cases of legal control), five cases of verified poaching, and finally, 18 cases of cryptic poaching (not included in the 26 verified mortalities). We considered a wolf as having been cryptically poached or verifiably poached according to criteria explained below. However, we could not obtain a robust estimate of cryptic poaching because we never found the supposedly dead wolves. We circumvented this obstacle by fitting a hierarchical state–space model to another dataset, a decade-long time series of population size and number of reproductions. In particular, we investigated whether the non-poaching mortality and the verified poaching rates would be large enough altogether to explain the observed population trends, or, on the contrary, if an additional source of mortality was needed to fit the longitudinal data the best.

### (b) Criteria for cryptic and verified poaching

Cryptic poaching was defined based on four criteria (with either all of criteria 1–3 or criterion 4 alone satisfied):

1. Sudden loss of radio contact with no indication of transmitter failure (more than half of the expected battery life-time remaining).
2. At least two aerial searches over a much larger area than the wolf territory were performed without further contact with the collared individual.
3. The individual was resident and repeated snow-tracking within the territory, in combination with the collection of scats and subsequent DNA analyses of multiple faeces confirmed that this individual was no longer present within the pack territory.
4. Radio contact was lost and special circumstances strongly indicated that poaching was the most plausible explanation. This applied only for two cases where police reports confirmed that people had attempted to poach wolves.

Wolves not satisfying these criteria were censored at the date of lost contact.

Verified poaching was defined based on two criteria (enough if one criterion is satisfied):

1. The body was recovered and the necropsy showed that a human deliberately killed it outside a legal hunt.
2. Wolf tissue (skin or muscle) determined by DNA analysis to originate from one of the radio-collared wolves was found in possession of a person that could not explain how he had acquired it and was later convicted at a court for this illegal possession.

### (c) Hierarchical model

To estimate the posterior distribution of the true size of the population, we composed process and observation equations. The process equation was
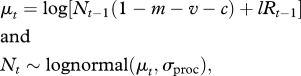
where *μ*_*t*_ is the deterministic prediction of the median wolf population size at time *t*, *N*_*t*_ is the true population size at time *t, *σ**_proc_ is the standard deviation of the true population size on the log scale, *m* is the mortality rate from all causes except poaching, *v* is the verified poaching rate, *c* is the cryptic poaching rate, *l* is the per pack recruitment rate and *R*_*t*_ is the number of reproductions at time *t*. The process equation was linked to data using the observation equation
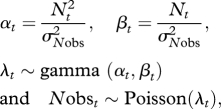
where *N*obs_*t*_ is the observed population size at time *t*, *σ*^*2*^_*N*obs_ is the estimate of the error of observation of the population size. This formulation views the count data hierarchically—the mean observed count of wolves at time *t* is Poisson distributed with mean *λ*_*t*_ and this mean is drawn from a gamma distribution with mean equal to the prediction of the process model and a standard deviation for observation error. We chose this approach because it allows the uncertainty in the data model to be larger than the variance of the Poisson parameter *λ*_*t*_. The approach is the same as assuming that the count data follow a negative binomial distribution, but offer computational advantages [17]. We did not include density- dependence in our hierarchical model because there is plenty of space and wild ungulate prey for larger wolf populations on the Scandinavian Peninsula, and both Sweden and Norway have some of the highest moose–wolf ratios in the world [18].

### (d) Data and model priors

Estimates of total population size (*N*obs) and number of reproductions (*R*) were obtained annually from 1999 to 2009, using a combination of snow-tracking, radio-tracking and DNA analysis of scats (see electronic supplementary material, time-series data).

Monitoring of pack reproductions provided informative prior on *l*. Number of pups at the age of six months was estimated from recurrent sessions of snow-tracking within territories (3.788 ± 1.466). Shape parameters of informative gamma-distributed prior for litter size *l* were then calculated using moment matching (table 1) [19].

**Table 1.** Parameter values from radio-tracking dataset and prior distributions. Priors shape parameters were derived by moment matching of mean and s.d. values from the radio-tracking dataset. Note that *σ*_proc_ is on a log scale.

parameter	mean value from radio-tracking	s.d.	prior
*m*	0.148	0.028	beta (23.44, 135.21)^a^
*v*	0.050	0.017	beta (7.84, 150.56)^a^
*c*	0.085	0.023	unif (0, 1)
*l*	3.788	1.466	gamma (6.67, 1.76)^a^
*σ*_*N*obs_	—	—	unif (0, 50)
*σ*_proc_	—	—	unif (0, 25)

^a^Denotes informative priors.

Using radio-tracking data, we calculated cause-specific mortality rates: non-poaching 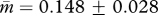, verified poaching 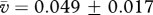, and suspected poaching 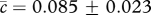, and accounting for competing risks [14]. Shape parameters of informative beta-distributed priors for rates *m* and *v* were calculated using moment matching (table 1) [19]. Although radio-tracking data could provide information also on cryptic poaching rate *c*, we specified an uninformative prior on *c* to investigate whether the observed population trends could be well explained with no cryptic poaching at all. While literature refers to cause-specific mortality rates, *c*, *m* and *v* are in fact probabilities to die from a specific cause, and therefore, the most uninformative prior we could give to *c* was a uniform distribution in (0,1).

Shape parameters for uninformative priors for *σ*_*N*obs_ and *σ*_proc_ were chosen subjectively to assure that all possible values in the posterior distribution had equal densities in the prior distribution (table 1). Sensitivity of posterior distributions to the priors was tested to assure the choices of shape parameters were uninformative.

### (e) Monte Carlo Markov Chain inference

We estimated the posterior distribution of each parameter by running Monte Carlo Markov Chains, implemented in JAGS [20] with R [21]. Six chains were initialized with different sets of parameter values chosen within biologically plausible bounds. After an initial burn-in period of 100 000 iterations, we obtained 1 000 000 iterations of each of the chains, thinning each by 10. We successfully checked for convergence using the Heidelberger & Welch [22] stationarity and half-width tests with the CODA package [23]. We evaluated the overlap between prior *p*(*θ*) and posterior *π*(*θ*|*y*) distributions by computing the quantity 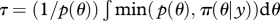 [24].

To estimate the impact of poaching on our study population, we simulated population trajectories from 1999 to 2009 using posterior distributions of parameters without cryptic poaching and without any poaching at all. We also wanted to investigate if we could differentiate an absence of cryptic poaching from small rates of cryptic poaching. For this, we considered that the simulated population without cryptic poaching would become our longitudinal data and we used the same approach to estimate the posterior distribution of cryptic poaching. This amounts to fitting a model to a dataset that we know has been generated with no cryptic poaching at all.

## 3. Results

**Figure 1.**
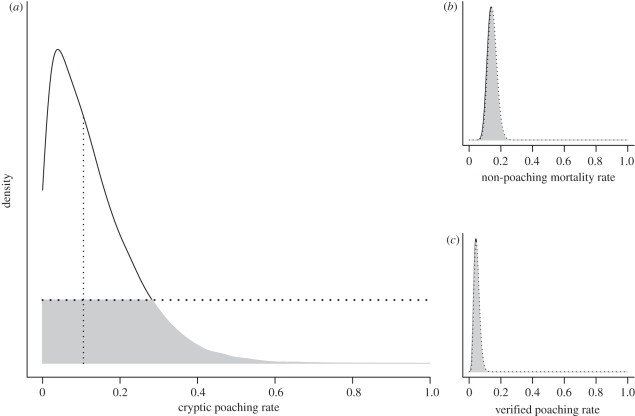
Posterior (solid black line) and prior (dotted black line) densities for (*a*) cryptic poaching rate *c* (posterior median = 0.103 ± 0.106, shown by vertical thin line), (*b*) non-poaching mortality rate *m* (posterior median = 0.142 ± 0.027) and (*c*) verified poaching rate *v* (posterior median = 0.046 ± 0.016). Overlap between prior and posterior densities is shown by the grey area. Parameters *m* and *v* were given informative priors based on radio-tracking data. Their posterior median estimates were very similar to rates from radio-tracking data (non-poaching mortality rate = 0.148 ± 0.028, verified poaching rate = 0.049 ± 0.017). The prior for cryptic poaching rate was on the contrary left uninformative. Still, its posterior median estimate was remarkably similar to the independent estimate of cryptic poaching rate from radio-tracking data (0.085 ± 0.023). The posterior density of cryptic poaching poorly overlapped with its prior and reveals that an unobserved source of mortality was present in the population.

Poaching accounted for half of total mortality (51%) and more than two-third (69%) of total poaching was cryptic. The median estimates of posterior non-poaching (0.142 ± 0.027) and verified poaching (0.046 ± 0.016) mortality rates from the model were very similar to the rates based on radio-tracking data (respectively, 0.148 ± 0.028 and 0.049 ± 0.017; figure 1). The median estimate of the posterior cryptic poaching rate from the model (0.103 ± 0.106) was also remarkably close to the independent estimate based on radio-tracking data (0.085 ± 0.023) but was accompanied by higher variance (figure 1). However, despite this variance, the data improved the estimate of cryptic poaching over prior knowledge. Overlap was large for non-poaching (*τ* = 94%) and verified poaching (*τ* = 96%) mortality rates. On the contrary, for cryptic poaching overlap was smaller (*τ* = 37%) indicating that modelling did add information on the estimate of cryptic poaching.

**Figure 2.**
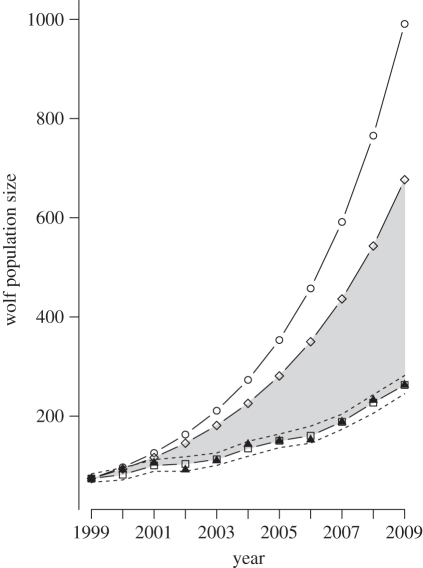
Model and census estimates of the wolf population in Scandinavia during 1999–2009. Filled black triangles are census data. Squares are median of posterior distribution of the fitted model, with its 95% credible interval shown by dashed lines. Circles are the median posterior distribution of the simulated population without poaching assuming no density-dependence. This reveals a decade of poaching scaled down population size from 990 to 263 wolves in 2009. Lozenges are the median posterior distribution of the simulated population without cryptic poaching assuming no density-dependence. The grey area indicates the number of wolves theoretically lost due to cryptic poaching.

Our study population increased from 74 individuals in winter 1998/1999 to 263 in 2008/2009 (figure 2). The mean annual growth rate during this period was 13.5 per cent. Assuming no (verified and cryptic) poaching and no density-dependence, this trajectory would have resulted in a median population size of 990 wolves in 2009, i.e. almost four times larger than the one observed. For a population without cryptic poaching and with verified poaching only, the trajectory would have resulted in a median population size of 676 wolves in 2009. When we considered the simulated population without cryptic poaching as data and used the same modelling approach to quantify cryptic poaching, we obtained a rate of cryptic poaching very close to zero (*c* = 0.023 ± 0.03).

## 4. Discussion

Here we have demonstrated a high incidence of poaching in a threatened wolf population, but because a major part of this poaching was unobserved (cryptic) and inferred from indirect data, its estimate is open to criticism. Although we cannot unequivocally prove that the posterior of rate *c* strictly includes only poaching, we can conclude that an additional source of mortality is required to explain our combined data. Because we could not identify any other cause of death than poaching that also would have resulted in a sudden loss of radio contact combined with no later verification through the continuous DNA-monitoring of the population, we believe that most, if not all, mortality included in this rate is indeed cryptic poaching. The close correspondence between the estimates we obtained for cryptic poaching rate from the model (0.103) and the independent one based on radio-tracking data (0.085) furthermore support that cryptic poaching indeed was an important mortality cause in our study population. The larger model estimate might be explained by the fact that the estimate based on radio-tracking data could be an underestimate. Because of our strict criteria for poaching, we did not classify any of the missing radio-collared non-resident dispersers as cryptic poaching, although this may have occurred in some cases. Our estimate of cryptic poaching received further support by the large gap between the simulated population trend without cryptic poaching and the observed dataset. This should convincingly reveal that observable mortality rates *m* and *v* cannot alone explain the observed population trends.

Our results may have been severely biased if we had underestimated population size, because, in that case, the parameter we attributed to cryptic poaching would actually have been a correction factor for our underestimate. However, the intense and continuous fine scale monitoring of wolves allowed us to rule out a systematic underestimate of population size (see electronic supplementary material, robustness of census data). Our results may have been equally biased if our criteria for cryptic poaching were inadequate. However, none of the 18 animals classified as cryptic poaching were ever detected after the loss of radio contact by any of the survey methods used in this study (see electronic supplementary material, robustness of poaching criteria). Quantifying a cause-specific mortality rate based on unknown fates requires also excluding the possibilities that an animal would have remained undetected by dispersing from the population. The breeding wolf population on the Scandinavian peninsula (Norway and Sweden) appears to be functionally isolated from the Finnish–Russian population with very little immigration and only one confirmed emigration recorded during the past decade (see electronic supplementary material, population isolation).

Poaching has had a significant impact on the population recovery. An average annual growth rate of 13.5 per cent is well below the typical rate of colonizing or recovering wolf populations [25]. Without any poaching, the median annual growth rate of our study population would have been 29.5 per cent during the period, i.e. more than double the observed rate and compatible with the fastest recovering wolf populations on record [25,26]. Considering that neither suitable habitat nor prey base are limiting factors, the population size in 2009 would probably have been three to four times the one observed (figure 2). Although the population has continued to grow, the decelerated growth rate caused by poaching is having other negative consequences. It has postponed the time when managing authorities can be more flexible with permits to kill problem individuals, causing unnecessary conflict with local people. Still more serious, it has aggravated an already bad genetic situation. The Scandinavian wolf population is small, isolated and facing serious genetic problems [27,28], and any delay in growth will accelerate inbreeding and loss of genetic variation [29].

Few studies of large carnivore survival based on radio-tracking have clearly described how they have treated cases of lost radio contact and made efforts to differentiate between possible fates of these animals. In a newly protected wolf population in north central Minnesota, a substantial proportion of lost radio contacts was assumed to be caused by illegal killing, and estimated to make up 70 per cent of total mortality rate [30]. In three different Scandinavian lynx (*Lynx lynx*) populations, formal criteria resembling the ones we have used in this study were set up for validation of each case of lost radio contact, resulting in poaching rates between 32 and 74 per cent of total mortality [31]. A study on wolverines (*Gulo gulo*) also differentiated among lost radio contacts, and estimated that poaching made up 60 per cent of total mortality [32], while in a study of Amur tigers (*Panthera tigris*), ratio of poaching to total mortality was 75 per cent [15]. A shared result in these studies was that a substantial part of the estimated poaching rates was made up of cryptic poaching (44–71%). Cryptic poaching was estimated to be 69 per cent of the total poaching rate in our study, which falls within the range of these earlier studies. Had all cases of lost radio contact just been censored from further calculations, both poaching and total mortality rates would have been seriously underestimated. However, as the estimates of the cryptic part of poaching in all these case studies were based on assumptions, the degree of uncertainty in the estimates was unknown. By using a hierarchical model, we could combine multiple sources of data in a statistically coherent way and unobserved quantities could be estimated because of their interdependence with the quantities that are observed.

We believe that the results presented above, motivate careful reconsideration of the extent of cryptic poaching in all studies of large carnivores. A recent example is the extensive study of survival in the newly recovered Northern Rocky Mountain wolf population in northwestern United States [33], where a minimum of 87 (24%) of the 363 dead animals was confirmed illegally killed. A further number of 150 animals were censored at the date of lost radio contact. Although the authors gave several arguments why it was less likely that these animals might have been poached, we caution that this indeed might be the case for a substantial part of them, especially considering that the study was performed in an extremely wolf hostile human environment.

We have shown that the failure to include the effects of cryptic poaching can cause serious errors in the estimation of the potential rate of population growth. Because a substantial part of poaching is often unobserved, poaching may be an even larger problem in wildlife conservation than has hitherto been assumed owing to the difficulty of measuring it properly. Quantifying cryptic poaching and its impact illustrates a challenging problem that is not unusual in ecology and conservation biology—the estimation of unobservable parameters with small values but high variance [34]. As we have illustrated here, such problem can be successfully addressed by combining multiple data in a hierarchical framework to obtain robust inferences. The increasing possibilities to mark many more individual animals at a much larger range of taxa, body sizes and length of tracking time [35,36] should make collecting individual data more feasible, and therefore, our approach more widely applicable in the future. Our study should further reinforce the need to bring uncertainty to the centre stage of conservation studies [37] and illustrate how considering uncertainty affects the ability to manage populations [3,38].
